# Offspring of parents with Balkan Endemic Nephropathy have higher C-reactive protein levels suggestive of inflammatory processes: a longitudinal study

**DOI:** 10.1186/1471-2369-10-10

**Published:** 2009-04-28

**Authors:** Wilfried Karmaus, Plamen Dimitrov, Valeri Simeonov, Svetla Tsolova, Vecihi Batuman

**Affiliations:** 1 Department of Epidemiology and Biostatistics, University of South Carolina, Columbia, South Carolina, USA; 2 National Center of Public Health Protection, Sofia, Bulgaria; 3 Vratza District Hospital, Vratza, Bulgaria; 4 Section of Nephrology-Hypertension, Tulane University Medical Center, New Orleans, USA

## Abstract

**Background:**

Despite the characteristic extensive tubulointerstitial fibrosis, Balkan Endemic Nephropathy (BEN) is usually considered a non-inflammatory disease.

**Methods:**

We examined a marker of inflammation, C-reactive protein (CRP), in the offspring of patients with BEN, a population at risk for BEN, prior to development of established disease to determine if an inflammatory process could be identified in the early stages of the disease. In 2003/04, 102 adult offspring whose parents had BEN and a control group of 99 adult offspring of non-BEN patients were enrolled in this prospective study. This cohort was re-examined yearly for four consecutive years. Levels of serum CRP were measured in years 3 and 4 and compared between groups. The data were analyzed with mixed models.

**Results:**

Compared to controls, offspring of BEN parents had statistically higher CRP levels in two consecutive years, suggestive of early inflammatory reactivity. Whenever the mother was affected by BEN (both parents, or mother only), serum CRP was significantly increased, but not if only the father had BEN. CRP was inversely related to kidney cortex width but not to markers or renal function.

**Conclusion:**

Early stages of BEN may involve inflammatory processes. The observation of a maternal involvement supports the concept of fetal programming, which has been implicated in the pathogenesis of other chronic kidney diseases.

## Background

Balkan Endemic Nephropathy (BEN) is a tubulointerstitial kidney disease which final stage is characterized by renal failure and shrinkage of both kidneys [1]. Cases of BEN were first described in Bulgaria in 1956 [2], then in Yugoslavia in 1957 [3], and in Romania in 1961 [4]. In 1964, this disease was recognized as a new nosological entity, although its causes are still unknown. The number of people diagnosed with BEN is at least 25,000, while approximately 100,000 persons are at risk [5]. Although this disease is endemic in rural areas of the Balkan, endemic nephropathies are reported to occur in other regions too. For instance, chronic kidney diseases of uncertain etiology, which resemble BEN, have been reported in Sri Lanka (personal communication, Dr. Alturaliya, Sri Lanka) [6]. In the past 50 years, etiologic explanations have emerged emphasizing for instance lignites or organic substances from coal [5,7], aristolochic acid [8], ochratoxin A [9], metals, and metalloids [10-13]. However, these claims are often not substantiated by sufficient evidence or scientific studies. For instance, we recently reported negative findings for metals and metalloids [14]. Or regarding aristolochic acid, there are no published scientific studies that provide biological evidence of association between aristolochic acid exposure and incidence or prevalence of BEN in humans [15,16]. To our understanding, there is no sound explanation of risk factors for BEN, only lack of knowledge.

Regarding the clinical features of the disease, a number of descriptions that emerged in the 1950s and 1960s also did not result from thorough scientific inquiries. Hence, we need scientifically sound investigations of basic assumptions. One is, that BEN develops without inflammation and that it progresses slowly over many years [17,18]. Because BEN is usually diagnosed in its late stage, there are few studies describing earlier stages of its development. However, this restriction limits evaluation on whether inflammatory mechanisms were involved in the beginning. Established BEN is characterized by marked tubulointerstitial fibrosis and tubule atrophy without evidence of significant inflammatory cell infiltration. This has been taken as evidence that BEN is not an inflammatory disease. However, it is hard to imagine how such marked tubulointerstitial destruction can occur without inflammation. We therefore decided to investigate CRP as a marker of inflammation in subjects at risk for BEN but without established disease, to determine if evidence for inflammation could be detected early during the course of BEN. CRP is an acute phase protein whose synthesis in the liver is regulated by different cytokines. Plasma levels of CRP in the absence of active disease are low, but can rise up to a thousand-fold in patients with an inflammatory reaction [19].

This became our motivation for a follow-up study investigating clinical markers of Balkan Endemic Nephropathy in a cohort at risk of the disease at an early stage. We recruited adult offspring of BEN patients and compared these with non-BEN offspring. Earlier, we demonstrated that kidney length and minimal cortex width in BEN offspring were significantly decreased if the mother had BEN [20]. Additionally, blood pressure, as well as urine concentrations of total protein, albumin and β2-microglobulin, were higher in the maternal BEN offspring [20,21]. However, using laboratory and clinical criteria, in 2006/07 none of the offspring had yet been diagnosed of having BEN. For this analysis, we hypothesized that adult offspring of BEN patients have increased levels of CRP, in particular if their mother had BEN. Because we detected significantly increased CRP levels in the 2005/06 investigations, we repeated the CRP analysis in 2006/07 to test whether we could substantiate our initial findings and whether this is an ongoing inflammatory state.

## Methods

### Population

From October 2003 to April 2004, we recruited 102 adult offspring whose father and/or mother were included in the Vratza Hospital registry of BEN patients in Bulgaria in 2001 and who resided in one of three communities (Vratza, Bistretz, and Beli Izvor in Bulgaria). The diagnosis of BEN in the parent generation was based on published criteria [22]. A control group of nearly equal size, 99 adult offspring of non-BEN hospitalized patients, was enrolled in the study during the same time period. Diagnoses in controls' parents included diabetes mellitus, and cardiovascular and liver disorders. Only three of the 99 controls had parents with kidney disorders (one paternal kidney cancer not related to BEN, and two maternal pyelonephritis cases). Subjects of both groups were frequency-matched according to gender and ten-year age groupings. In the third examination (2005/06), a parent of one control participant had developed BEN [20,21]. Thus, this participant was moved into the BEN offspring group for analyses of this and subsequent years.

All participants provided written consent through a procedure approved by the Institutional Review Board (Human-Subject Research Committee) of the National Center of Public Health Protection, Sofia, Bulgaria. This population was re-examined in 2004/05, 2005/06, and 2006/07.

### Interviews

We conducted face-to-face interviews with all participants either in the hospital, or by visiting them in their home village. The standardized questionnaire included their family history of BEN and of other kidney diseases.

### Physical examination and CRP measurement

Physical examinations were performed by an experienced physician with board certifications in internal diseases and nephrology. No patient showed clinical signs of inflammation or infection during the study period. Blood pressure was measured according to protocols established by the World Health Organization [23]. Venous blood for the determination of CRP was drawn in K_2_EDTA Vacutainers^® ^in the third and fourth investigation in 2005/06 and 2006/07. Blood samples were centrifuged and serum CRP was measured with IMMULITE^® ^chemiluminescent immunometric assay. The expected value for healthy volunteers is 0.14 mg/dL and the upper 97.5 percentile is 1.1 mg/dL.

To determine whether CRP levels are related to having a parent with BEN, we compared CRP levels in the offspring of these two groups. Second, to determine whether maternal or paternal history of BEN was involved, we grouped parental disease status into four categories: mother, father, both parents, and none affected. The comparison offspring included only parents not affected by BEN (reference group). As CRP was not normally distributed, we applied non-parametric tests (Kruskal-Wallis) for descriptive purposes.

### Statistical Analyses

The repeated measurements in 2005/06 and 2006/07 were not independent, hence, we used mixed models (PROC MIXED) adjusting for within-participant effects [24]. We used the regular maximum likelihood method of estimation. Linear mixed models require that the random effects and the error vector were normally distributed. To achieve this, we log-transformed the CRP values, thereby providing geometric means for the various risk factors in the CRP explanatory model. For the within-subject association, we used an unstructured covariance model, which requires the least amount of constraints. For the repeated covariance structure, the variance component provided the best fit (Akaike Information Criterion). We performed data analyses using SAS version 9.1 (SAS Institute Inc., Cary, NC, USA).

The two risk factors of interest were parental history of BEN (yes vs. no) and a four-level categorization of this variable: mother, father, both parents, and none affected. Statistically, we controlled for gender, age, smoking in the last 12 months, ex-smoking, body-mass index (BMI: kg/m^2^), history of diseases of the urogenital system, diabetes mellitus, and medication. For each year, based on their history, participants were categorized into active smokers and ex-smokers. Statistically we controlled for the following urogenital diseases in the offspring: cystitis, pyelonephritis, kidney stones, and other kidney diseases (e.g., cancer: n = 2, and hydronephrosis n = 1). In addition, we treated diabetes in the participant as a potential confounder, since it can affect both the kidneys and inflammatory markers. To classify diabetes, we used information provided by the participant in addition to serum glucose measurements. Once participants stated that they had diabetes in one of the four years, they were classified as diabetic for that and the subsequent year(s). If serum glucose was higher than 6.2 mmol/L in at least two years, we also classified the participant as diabetic. Regarding medication, we adjusted for anti-diabetes drugs (thiazolidinediones and others), beta-blockers, and steroid hormones.

## Results

C-reactive protein was determined in the third and fourth-year follow-up: 2005/06 and 2006/07. Of the initial 201 participants (2003/04), 182 participated in the third investigation (90%) in 2005/06. In addition, 18 new offspring of BEN parents were recruited in 2005/06 (total of 200 participants, table 1). In year 3, the father of one control participant developed BEN. Thus, this participant was re-allocated. In year 4, 193 participants were included. There were no significant differences in the distributions of gender, age, smoking, and body mass index in the offspring of BEN and the control group. Pyelonephritis, kidney stone, and use of beta-blockers were reported more frequently in the offspring of BEN patients.

**Table 1:** Population characteristics of the follow-up study on Balkan Endemic Nephropathy

		Investigation in 2005–2006		Investigation in 2006–2007	
		Offspring of BEN cases	Offspring of control patients	Offspring of BEN cases	Offspring of control patients
		n = 106 %	n = 94 %	n = 101 %	n = 92 %
Parental history of BEN	Both	30.2	0	30.7	0
	Mother only	32.1	0	32.7	0
	Father only	37.4	0	36.6	0
	none	0	100	0	100
Gender	male	47.2	46.8	47.5	46.7
Age (years)	≤ 40	11.3	21.3	8.9	19.6
	40–50	35.9	34.0	35.6	34.8
	> 50–60	34.0	29.8	34.7	30.4
	60 & older	18.9	14.9	20.8	15.2
Smoking in the last 12 mths		37.5	38.3	35.6	35.9
Ex-smoking		21.6	10.4	16.8	13.4
History of: pyelonephritis		8.5	2.1	8.9	2.2
kidney stones		9.4	6.4	11.9	5.4
cystitis		5.7	6.4	5.9	6.5
other kidney diseases		3.8	2.1	5.9	2.2
Diabetes mellitus		2.8	7.5	5.9	8.7
Report of application of the following medications: Anti-diabetics		0.9	2.1	2.0	2.2
Beta-blockers		8.5	1.1	7.0	2.2
Steroid hormones		6.0	0	0	2.2

		Median (5 – 98% value)			
Age	(years)	51 (38–65)	48 (34–65)	52 (39–65)	49 (34–66)
Body-mass index	(kg/m^2^)	28.0 (19.5–35.7)	26.3 (21.4–33)	28.5 (19.8–35.0)	27.2 (21.5–34.3)

Due to a communication error with the laboratory, CRP was only determined in 141 of the enrolled participants; in 77% of those with parental BEN, and in 78% of adults without parental history of BEN. In the fourth year, we had 190 CRP measurements from 200 participants (table 2). Unadjusted median levels of CRP in the offspring of BEN parents were 0.56 mg/dL (5–95% values: 0.07–2.92 mg/dL) and 0.29 mg/dL in controls (5–95% values: 0.06–2.04 mg/dL, p < 0.001). In both measurement periods, whenever the mother was affected by BEN (either both parents or maternal history) serum CRP was significantly higher (table 2). This was not seen if only the father had a history of BEN. CRP levels in the two consecutive years showed a moderate and statistically significant rank correlation (r_Spearman _= 0.586, n = 151, p < 0.0001).

**Table 2:** Concentration of C-reactive protein and in adult offspring and parental history of BEN^#^

Variable	Parents had BEN			No parent affected								
	n	Median	5–95% value	n	Median	5–95% value						
C-reactive protein (mg/dL) 2005/06	85	0.56	0.07–2.92	74	0.29	0.06–2.04						
p-value		0.0028			reference							
C-reactive protein (mg/dL) 2006/07	99	0.36	0.07–3.41	91	0.22	0.05–1.66						
p-value		0.004										

	Both parents had BEN			Mother had BEN			Father had BEN			No parent affected		
	n	Median	5–95% value	n	Median	5–95% value	n	Median	5–95% value	n	Median	5–95% value

C-reactive protein (mg/dL) 2005/06	23	0.88	0.07–4.53	31	0.68	0.15–2.92	31	0.33	0.05–1.36	74	0.29	0.06–2.04
p-value		0.001			0.003			0.65			reference	
C-reactive protein (mg/dL) 2006/07	31	0.47	0.09–4.03	36	0.41	0.06–3.41	32	0.30	0.06-.171	91	0.22	0.05–1.66
p-value		0.007			0.015			0.61			reference	

# p-values based on Kruskal-Wallis test.

In repeated measurement models, being a BEN offspring was significantly associated with higher CRP levels (table 3). In addition, a maternal history of BEN (mother and/or both parents) was related to a higher CRP. Other important risk factors for higher CRP levels were age, increased body mass index, and smoking. Table 4 shows the estimated geometric means for CRP. When the mother had BEN, CRP is 0.58 mg/dL, and with both parents CRP is 0.64 mg/dL. There is no difference between control and paternal BEN (0.42 and 0.43 mg/dL). The descriptive (table 2) and the repeated measurement analyses (table 4) thus show comparable findings.

**Table 3:** Effect of being an offspring of a BEN patient on the level of C-reactive protein^#^

	Repeated measurement-analysis: 200 participants, 349 observations			
	Model comparing offspring BEN status		Model comparing parental history of BEN	
	Parameter Estimate^¥^	p-value (t-test)	Parameter Estimate^†^	p-value (t-test)
Offspring of BEN patient vs offspring of control patient	0.102	0.043	-	
Parental history of BEN:				
both	-		0.189	0.01
mother	-		0.146	0.03
father	-		0.013	0.85
none	-		Reference	
Age (years)	0.012	< 0.0001	0.01	0.0005
Gender (female)	0.073	0.178	0.06	0.26
Body-mass index	0.038	< 0.0001	0.038	< 0.0001
Smoking in the last 12 months	0.157	0.007	0.153	0.009

^# ^CRP (mg/dL) is log 10 transformed

^¥ ^Statistically controlled for ex-smoking, history of pyelonephritis, cystitis, other kidney diseases

† Statistically controlled for ex-smoking, history of cystitis, administration of steroid hormones

**Table 4:** Adjusted geometric means for C-reactive protein comparing offspring status and parental history of BEN^†^

	C-reactive protein (mg/dL) Repeated measurement analysis (200 participants, 349 observations)			
	Adjusted geometric mean	5–95% confidence limit		p-value (F-test)
Offspring of BEN patient	0.62	0.41	0.94	0.04
Offspring of control patient	0.49	0.31	0.77	
Parental history of BEN:				
both	0.64	0.41	1.0	0.026
mother	0.58	0.37	0.91	
father	0.43	0.27	0.68	
none	0.42	0.28	0.63	

† Statistically controlled for ex-smoking, history of cystitis, administration of steroid hormones

During the course of examinations over three years (2003/04, 2004/05, 2005/06) CRP was not systematically correlated with excretion of total protein, albumin, beta2-microglobulin, creatinine clearance (Cockcroft and Gault), nor with kidney length (data not shown, for methods see [20]). However, we found that CRP was inversely related to kidney cortex width, determined by ultrasound measurements (table 5). The negative correlation increased over the course of the investigations. No ultrasound measurements were conducted in 2007/08, the latest year of CRP measurements.

**Table 5:** Correlation coefficient of C-reactive protein with minimal width of the kidney cortex

	Kidney cortex width (mm) in 2003/04	Kidney cortex width (mm) in 2004/05	Kidney cortex width (mm) in 2005/06
Mean [n]	15.7 [n = 201]	15.4 [n = 189]	15.6 [n = 200]
Rank correlation with:			
C-reactive protein (mg/dL) 2005/06	-0.09† p = 0.32Δ n = 141Φ	-0.16 p = 0.058 n = 141	-0.26 p = 0.001 n = 159
C-reactive protein (mg/dL) 2006/07	-0.08 p = 0.30 n = 173	-0.19 p = 0.014 n = 141	-0.20 p = 0.006 n = 190

† rank correlation coefficient, Δ p-value, Φ number of observations

## Discussion

Our results show that being an offspring of BEN parents is associated with increased CRP background levels in two consecutive years, indicative of an inflammatory state in the adult offspring. Interestingly, a maternal history of BEN is a significant risk factor for higher background CRP levels in offspring, but paternal BEN is not.

Our findings are unlikely to have resulted from selection bias, since the retention of this cohort is high. It is also unlikely that the results are chance findings, since we found the same results for CRP in two consecutive years. CRP serum concentrations in our sample of healthy adults were within the range reported by other investigations [25,26]. We did find the classical risk factors (age, body mass index, and smoking) related to CRP [27,28]. There are numerical differences between the 2005/06 and 2006/07 measurements; the values in 2005/06 were higher. The variations may be due to different individual situations, season, infections, or measurement errors. However, despite these variations, the described effects in the four groups remained stable in two consecutive years. In addition, we also investigated whether the prior or current occupational status affected CRP levels or the association between parental history of BEN and CRP. We found that employees in the agricultural or transport sector had higher CRP levels (data not shown). Since these occupations were not differentially distributed among BEN and control offspring, the occupational status did not confound the association between paternal history and CRP.

Based on the paucity of inflammatory cell infiltration in kidney biopsy specimens, it has generally been thought that the extensive fibrosis and tubular atrophy seen in BEN is not an inflammatory process. Tatu *et al*., reported that levels of inflammatory cytokines and CRP were normal in most BEN cases [7], which would support the position that BEN is not an inflammatory disease, but their results remain unpublished. In contrast, in earlier presentation including 11 BEN patients, Dimitrov *et al*. reported increased erythrocyte sedimentation rate, a non-specific measure of inflammation[29] Also Toncheva *et al*. suggested that immune-mediated inflammatory processes may be involved in some patients [30]. Interestingly, CRP levels were inversely correlated with the kidney cortex width measured by ultrasound (table 5), but not with kidney length or other measured renal functions. It is possible, that CRP as an inflammatory marker is related to a reduction in the cortex width due to atrophy and interstitial scarring.

We found that a maternal history of BEN was a risk factor for increased CRP in the offspring. Earlier, we documented that the offspring of BEN parents had higher systolic and pulse pressure [21]. In addition, in this analysis we found that CRP levels are significantly correlated with systolic blood pressure (Spearman correlation coefficient: 0.33, p < 0.001 in 2005/06). This result is in agreement with prior reports that CRP is a risk factor for hypertension [20,25]. We believe that our findings emphasize the need for rigorous clinical-epidemiologic research to determine features and identify the inflammatory mechanisms of the disease that are comparable to other groups of renal pathologies.

Why could a maternal history of BEN be associated with a higher risk of BEN? We speculate that maternal conditions, for instance antibodies against kidney tissue, during pregnancy, at an age before the mother is diagnosed with BEN, may alter the development and the susceptibility of the offspring kidneys. The concept of prenatal programming may provide a new explanation for the observed family disposition of BEN.

This suspicion is further supported by prior findings showing that kidney size and function and blood pressure were also more strongly associated with a maternal history of BEN[20,21] However, this does not exclude the fact that environmental factors may have initiated and also sustained the maternal-offspring interaction. The idea that fetal programming is involved in BEN, [31] is further corroborated by research showing that fetal programming may also be involved in the pathogenesis of other chronic kidney diseases [32-34] Since cases of Balkan Endemic Nephropathy are registered and easily accessible in various Balkan countries, BEN may serve as a future model for determining the role of fetal programming in renal health.

## Conclusion

We found that offspring of patients with Balkan Endemic Nephropathy have higher CRP serum levels than offspring of parents without the disease. This finding suggests that inflammatory processes are involved in the pathogenesis of BEN. The maternal contribution to the susceptibility to BEN lends support to the evidence that prenatal programming may instigate a higher risk for developing BEN. Since two-generation studies can easily be established for Balkan Endemic Nephropathy, investigations into BEN provide an opportunity to characterize prenatal programming of kidney diseases in general.

## Competing interests

The authors declare that they have no competing interests.

## Authors' contributions

WK, PD and ST have contributed to developing the protocol of the medical exams. VS conducted the examinations and collected the blood samples; PD monitored the assays. WK, PD, ST, VS, and  VB considered and revised the analytical plan. WK and PD analyzed the data. All the authors contributed to and approved the final manuscript.

## Pre-publication history

The pre-publication history for this paper can be accessed here:

http://www.biomedcentral.com/1471-2369/10/10/prepub
